# Work out of office: how and when does employees’ self-control influence their remote work effectiveness?

**DOI:** 10.3389/fpsyg.2023.1265593

**Published:** 2023-10-18

**Authors:** Lei Qi, Yuping Xu, Bing Liu

**Affiliations:** ^1^School of Business Administration, Shandong University of Finance and Economics, Jinan, China; ^2^School of Management, Shandong University, Jinan, China

**Keywords:** self-control, remote work effectiveness, remote work self-efficacy, social support, social cognitive theory

## Abstract

**Introduction:**

The purpose of this study is to understand the positive effects of employees’ self-control on their self-efficacy and work effectiveness in the context of remote work, as well as social support (organizational support, interaction with supervisors, and family support) moderating role on such positive effects.

**Methods:**

Based on social cognitive theory, this study collects two-phase data with a sample of 240 remote workers.

**Results:**

The results show that employees’ self-control positively influences their remote work self-efficacy, which in turn positively increases their remote work effectiveness. Moreover, perceived organizational support, interaction with supervisors, and family support strengthen the effect of self-control on remote work self-efficacy.

**Discussion:**

First, this study explores the mechanism of self-control on remote work effectiveness, highlights the importance of self-control in remote work, and provides guidance for employees to improve remote work effectiveness. Second, this study discusses the mediating role of remote work self-efficacy between self-control and remote work effectiveness and reveals the psychological mechanism of employees’ self-control in remote work. Finally, this study comprehensively considers three types of support from work and family and analyzes the interaction between internal control and external support on remote work self-efficacy, which provides suggestions for enhancing employees’ confidence in remote work.

## 1. Introduction

With the rapid development of information technology, remote work has gradually become a common office mode (Karis et al., 2016; Stiles and Smart, 2021; Chatterjee et al., 2022; Howe and Menges, 2022). However, the outbreak of the COVID-19 pandemic rapidly advanced the development of remote work, transforming it from optional to mandatory (Becker et al., 2022; Jamsen et al., 2022). Given some benefits remote work has brought, many organizations plan to increase the amount of remote work and make it the norm in the post-pandemic era (Dwoskin, 2020; Chatterjee et al., 2022; Shifrin and Michel, 2022). It cannot be ignored that remote work inevitably brings a series of problems, such as reducing employees’ engagement, increasing management difficulty, and blurring work–family boundaries (Oksa et al., 2021; Syrek et al., 2022). After reviewing extensive literature, we argue that these problems reflect the low self-control of employees in remote work (Ghislieri et al., 2022; Troll et al., 2022). Self-control refers to the ability of individuals to actively adjust their thoughts, emotions, and behaviors to match their values and social expectations (Muraven and Baumeister, 2000; Vohs and Baumeister, 2004). Abundant evidence suggests that individuals with high self-control are better able to manage their thoughts and emotions, effectively inhibit negative behaviors, and exhibit higher job achievement than those with low self-control (de Ridder et al., 2012; Converse et al., 2018). Self-control is particularly important in remote work, where employees are far from the control and supervision of managers (Howe and Menges, 2022; Walsh et al., 2023). Most existing studies examined the influence of organizational and team factors, such as organizational support (Makikangas et al., 2022), human resource practices (Li et al., 2023), and internal communication (Dhanesh and Picherit-Duthler, 2021), while ignoring the importance of employees’ factors in remote work. Changes in the work environment and styles bring great challenges to employees’ cognition and behavior. As a result, they have to adopt self-control to monitor and manage their behavior (Maden-Eyiusta and Alparslan, 2022). However, the empowerment process that begins with self-control and leads to increased remote work effectiveness is understudied and deserves to be explored in depth (Goldsby et al., 2021).

The sudden shift to remote work brought a variety of challenges to employees in maintaining work effectiveness (Jamsen et al., 2022), most of which are related to low self-control (Troll et al., 2022; Uziel et al., 2022). According to Jex (1998), remote work effectiveness refers to the evaluation of remote workers’ work performance, which reflects the impact of their behavior on organizational goals. As the most essential problem in remote work, the improvement of remote work effectiveness is becoming increasingly important for enterprises (Grant et al., 2013; Adekoya et al., 2022). However, Zacharakis and Loos (2020) reported that many employees reported low work effectiveness during remote work. Discussions on remote work effectiveness were scarce, with few studies suggesting that flexible work preferences, smart work practices, and leadership roles may be contributing factors to remote work effectiveness (Wehrt et al., 2020; Adekoya et al., 2022). Although self-control plays an important role in remote work, existing research lacked a discussion of the relationship between self-control and remote work effectiveness (Lin, 2011). Based on this gap in the existing research, we explore how self-control affects remote work effectiveness to deeply analyze the promotion path of remote work effectiveness.

According to social cognitive theory, when people are exposed to the environment, they will exert their subjective initiative (Bandura, 2001). People tend to control and motivate themselves to keep striving to achieve their goals (Chatard and Seimbegovic, 2011). During this process, people form higher self-efficacy about their ability to achieve their goals, which in turn has an impact on their behavior and motivation (Bandura, 1978). Remote work self-efficacy refers to employees’ speculation and judgment on their ability to complete remote work tasks effectively (Staples et al., 1999). Compared with those who are strictly supervised in the traditional office mode, the self-efficacy judgment of remote workers may play a more important role in their work adaptation and task completion (Chang et al., 2021; Howe and Menges, 2022). Previous studies explored the antecedents and consequences of remote work self-efficacy, and results showed that many of the antecedents could be controlled and managed, such as remote work experience and training, information technology experience, and computer anxiety (Staples et al., 1999). However, although some scholars pointed out that self-control can help employees resist distractions and temptations and positively affect employees’ remote work self-efficacy (Staples et al., 1999; Troll et al., 2022), there is a lack of relevant empirical research. Evidence indicated that remote work self-efficacy has a positive impact on employees’ job performance, job satisfaction and organizational commitment (Staples et al., 1999; Raghuram et al., 2003; Guay et al., 2020). Existing research suggests that when employees are away from the workplace, they can develop stronger remote work self-efficacy in the process of self-control, which in turn affects their work outcomes (Straus et al., 2022), these findings also provide a basis for us to explore the important role of self-efficacy in remote work. While the mediating role of remote work self-efficacy between self-control and work outcomes has not been fully explored. Thus, based on social cognitive theory, we will reveal the mediating role of remote work self-efficacy between self-control and remote work effectiveness.

The basic view of social cognitive theory is that human activities are determined by the interaction of individual behavior, individual cognition, and other characteristics, and the external environment in which individuals live (Bandura, 2001). Combined with previous studies, we find that the effectiveness of self-control is easily influenced by the situation (de Ridder et al., 2012). As an important source of employee perceptions of support, support from work and family can complement employees’ self-control and meet the needs of employees to complete their work tasks (Baumeister and Exline, 1999; Chen et al., 2021; van Zoonen et al., 2021). For remote workers, organizational support, interaction with supervisors, and family support are important external assistance, which can increase employees’ confidence in completing work tasks, thus better exerting their self-control (Orkibi et al., 2018; Chen et al., 2021; Jamsen et al., 2022). Perceived organizational support refers to employees’ subjective perception that the organization values their contributions and cares about their well-being (Eisenberger et al., 1986). Interaction with supervisors refers to the communication and exchange between subordinates and supervisors inside and outside of work for mutual assistance and benefit (Law et al., 2000). Family support refers to the practical help and emotional support that employees get from family members (Mehreen and Ali, 2022). Existing studies have explored the influence of work and family support on employees’ cognition and behavior in the traditional office mode, while ignoring its role in remote work (Barnett et al., 2019; Kalliath et al., 2019). In remote work, employees have a low sense of participation and belonging and believe that they have limited external support (Hafermalz and Riemer, 2021; Kakkar et al., 2022). In this case, remote workers will regard support from work and family as an important complement and enhance their confidence in completing remote work tasks (Barnett et al., 2019; Chen et al., 2021). Based on the above considerations, we will explore the moderating effects of organizational support, interaction with supervisors, and family support between self-control and remote work self-efficacy.

**Figure 1:**
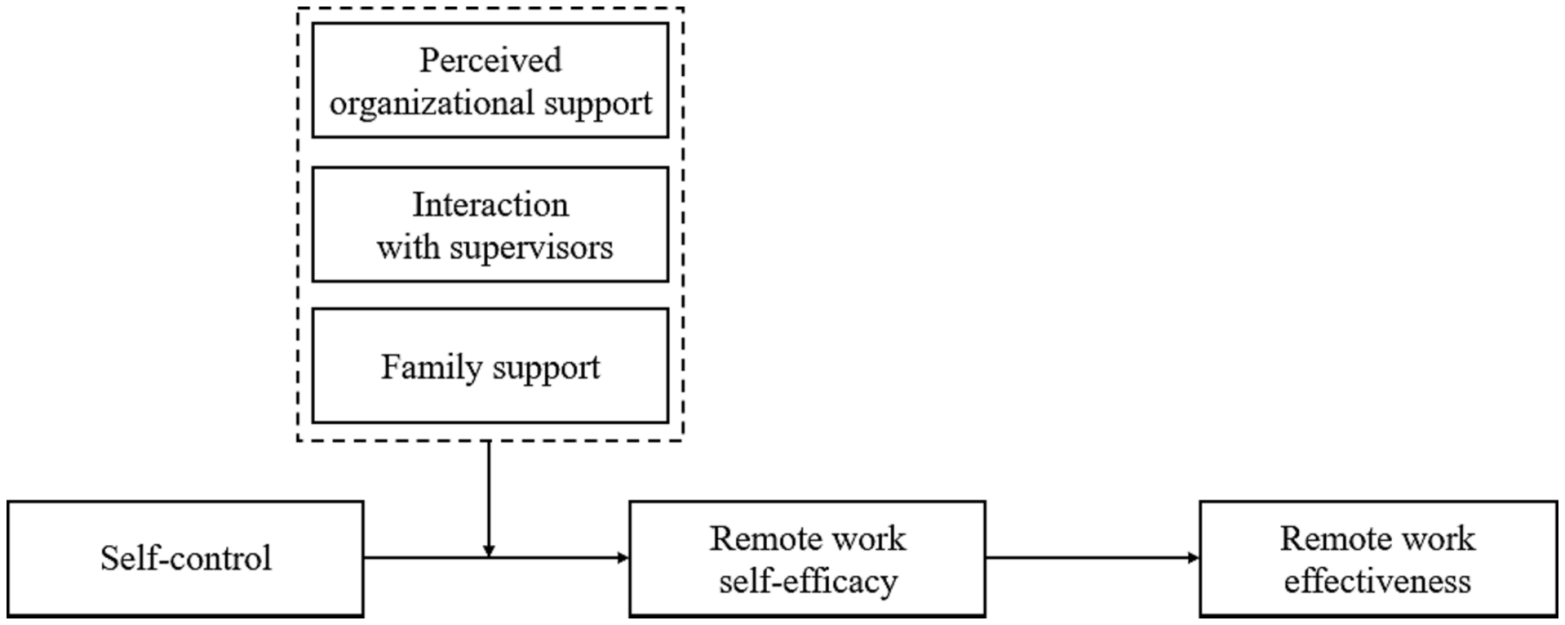
Hypothesized research model.

The current study contributes in three ways. First, we emphasize the important role of self-control in remote work and consider the ways to improve remote work effectiveness from the perspective of employees. Existing research has done a lot of work on the improvement of remote work effectiveness, while mainly focusing on the organizational and team level (Dhanesh and Picherit-Duthler, 2021; Makikangas et al., 2022), the employee factors have been neglected (Podolsky et al., 2022). As a change in the office mode, remote work not only brings challenges to employees but also highlights the important role of employees’ self-control (Maden-Eyiusta and Alparslan, 2022). We explore and test whether and how self-control affects employees’ remote work outcomes, which enrich individual-level influencing factors of remote work effectiveness. Second, we examine the mediating role of remote work self-efficacy between competence and performance and demonstrate the motivational processes that self-control leads to increased employee remote work effectiveness. Social cognitive theory emphasizes the necessity of self-efficacy research in specific situations (Bandura, 2001), while remote work self-efficacy has not received sufficient attention. Remote workers are far from the traditional workplace, and their self-efficacy acquired through self-control in remote work is more prominent than which in traditional work (Chang et al., 2021). We discuss the psychological mechanism of employees’ self-control in remote work and reveal the role of remote work self-efficacy in connecting employees’ self-control and remote work outcomes. Finally, we pay attention to the role of specific work and family supports in the context of remote work and analyze how external support factors shape work outcomes of employees’ self-control. Previous studies focused on the influence of various supports in the traditional office mode (Barnett et al., 2019; Chen et al., 2021), which was not discussed in remote work. Remote work keeps employees away from the traditional workplace and reduces their sense of identity and belonging. Support from work and family can provide material and spiritual help and supplement for employees, which is very important for remote workers who need self-control (Chen et al., 2021). Our study comprehensively considers three types of support from work and family and analyzes the interaction between internal control and external support on remote work self-efficacy, which provides suggestions for enhancing employees’ confidence in remote work. Figure **1** depicts the research model for this study.

## 2. Theory and hypotheses

### 2.1. Self-control and remote work self-efficacy

Self-control can trigger or inhibit specific behaviors, which is considered a balance mechanism between internal motivation and external demands (Hofmann et al., 2009; Fan et al., 2020; Li et al., 2020). Studies have shown that self-control enables individuals to better suppress impulses, resist temptations, and regulate emotions (Clinton et al., 2020) thus increasing work achievements and improving interpersonal relationships (Fan et al., 2020). A recent study also emphasized the importance of self-control in remote work (Troll et al., 2022). Given the beneficial influence of self-control on individual behavior, we attempt to apply it in the current study to reveal its relationship with employees’ cognition and behavior during remote work.

Self-efficacy is developed on the basis of social cognitive theory, which refers to individuals’ judgment on their ability to perform specific behaviors (Bandura, 1978). To conduct an in-depth study on remote work, Staples et al. (1999) proposed the concept of remote work self-efficacy, which referred to employees’ speculation and judgment on their ability to effectively complete remote work tasks. According to social cognitive theory, the formation of self-efficacy is mainly affected by four factors (Bandura, 1978): First is direct experience, the individuals’ assessment of their mastery achievement (Silver et al., 1995). Second is vicarious experiences, one’s judgment of self-efficacy may be formed by observing the behavior of others (Gist and Mitchell, 1992). Third is verbal persuasion—encouragement, evaluation, and suggestions from others (Bandura and Cervone, 1986). Fourth is one’s physical and mental state, namely the physiological or emotional state of the individual (Bandura, 1978). Because different situations provide different information to individuals, their cognition of self-efficacy will change with specific situations (Bandura, 2001).

We infer that self-control enhances employees’ remote work self-efficacy in the context of remote work. First, from the perspective of direct experiences, individuals with a strong capacity for self-control have higher expectations that events will have positive results and can accumulate more successful experiences in remote work (Hankonen et al., 2014). These successful experiences make employees confident in their remote work ability and enhance their remote work self-efficacy (Converse et al., 2018; de Ridder et al., 2020). Second, from the perspective of physical and mental state, employees with strong self-control capacity can timely self-regulate and better manage their emotions and behaviors (Nielsen et al., 2019; Clinton et al., 2020). A good physical and mental state can guide remote workers to self-motivate, strengthen self-identity, and generate strong remote work self-efficacy. Studies showed that remote workers who respond to challenges with a more positive attitude strengthen their self-motivation and thus exhibit higher remote work productivity (Howe and Menges, 2022). Therefore, we propose the following hypothesis:

Hypothesis 1: Self-control is positively related to remote work self-efficacy.

### 2.2. Remote work self-efficacy and remote work effectiveness

According to Schuler and Jackson (1987), there are three indicators for assessing work effectiveness: productivity, quality, and innovation. Given its importance in organizations, employees’ work effectiveness has been a constant concern of practitioners and scholars (Adekoya et al., 2022). With the development of remote work, scholars have explored the antecedents of remote work effectiveness (Grant et al., 2013). Kowalski and Ślebarska (2022) analyzed predictors of remote work effectiveness from the perspective of leaders and found that benefits (i.e., available communication devices, on-task concentration, and work economy), limitations (i.e., lack of rules, poor communication, and decreased work productivity) affect the remote work effectiveness perceived by managers. At the same time, the study calls for future research to explore the impact of employees’ attributes or other factors on remote work effectiveness from their perspective. In addition, some research showed that several key competencies may contribute to remote work effectiveness, including self-confidence, self-motivation, and good communication skills (Grant et al., 2013).

We argue that remote work self-efficacy will improve employees’ remote work effectiveness. Based on social cognitive theory, self-efficacy works through four processes of individual choice: selection, cognition, motivation, and emotion (Bandura, 1978). First, from the perspective of selection process, remote workers with high self-efficacy have higher expectations regarding their work outcomes, are open to meeting challenges at work, and are more likely to engage in innovative activities (Howe and Menges, 2022). Second, from the perspective of cognitive process, employees with high remote work self-efficacy tend to focus on how to control the current task and set reasonable goals at work, which can ensure the quality of their remote work (Raghuram et al., 2003; Howe and Menges, 2022). Third, from the perspective of the motivation process, employees with high remote work self-efficacy put more effort and persistence into their work tasks, which is conducive to improving remote work productivity (Howe and Menges, 2022). Finally, from the perspective of emotional processes, when faced with stress in remote work, employees with high self-efficacy can deal with them rationally and use positive emotions to cope with difficulties, which enhances remote work effectiveness (Makikangas et al., 2022). It has been shown that remote workers with high self-efficacy can better adapt to remote work and complete work tasks effectively (Staples et al., 1999; Howe and Menges, 2022). Therefore, we posit the following hypothesis:

Hypothesis 2: Remote work self-efficacy is positively related to remote work effectiveness.

### 2.3. The mediating role of remote work self-efficacy

Bandura (1978) argued that self-efficacy is the result of an individual’s evaluation of his or her abilities, which in turn regulates an individual’s behavior and affects his or her performance in specific tasks. Combining Hypothesis 1 and Hypothesis 2, we further speculate that self-control will have an indirect effect on remote work effectiveness through remote work self-efficacy.

First, employees with high self-control have greater mastery of remote work and can actively face challenges at work (Clinton et al., 2020; Fan et al., 2020), leading to higher remote work self-efficacy (Howe and Menges, 2022; Makikangas et al., 2022). When employees have a sufficient grasp of their ability to complete remote work tasks, they are willing to make more effort, which contributes to remote work effectiveness (Straus et al., 2022).

Second, employees with high self-control can persist in completing work tasks and avoid target deviation or task interruption, which is crucial to the realization of remote work goals (Converse et al., 2018). The smooth implementation of remote work will increase employees’ confidence, which in turn will facilitate the achievement of work goals (Howe and Menges, 2022). Previous research proved our inference by stating that successfully coping with challenges and completing tasks will enhance employees’ remote work self-efficacy, which in turn facilitates the improvement of employees’ engagement to ensure remote work effectiveness (Straus et al., 2022; Troll et al., 2022). With these points in mind, we argue for the following:

Hypothesis 3: Remote work self-efficacy mediates the relationship between self-control and remote work effectiveness.

### 2.4. The moderating effect of perceived organizational support, interaction with supervisors, and family support

As an important indicator of employees’ evaluation of the organizational environment, perceived organizational support can influence employees’ intrinsic and extrinsic motivation to achieve organizational goals (Li et al., 2018). The higher the perceived organizational support, the more willing employees are to contribute to the development of the organization (Chatterjee et al., 2022). Previous studies have found that perceived organizational support is usually associated with positive job outcomes, such as higher job performance, job satisfaction, and job engagement (Li et al., 2018; Firmansyah et al., 2022), as well as effectively promoting employees’ positive emotions and behaviors and inhibiting their negative reactions (Chiang and Hsieh, 2012; Sang et al., 2017).

We suggest that perceived organizational support moderates the relationship between self-control and remote work self-efficacy. Based on social cognitive theory, encouragement and evaluation of others is an important source of employees’ self-efficacy (Bandura, 1978). From the perspective of verbal persuasion, when perceived organizational support is high, remote workers perceive that the organization has expressed recognition and trust in their work ability (Zhang et al., 2021), especially in the context of remote work, where employees are far removed from offices. Attention from the organization increases employees’ affirmation of their capabilities and generates stronger remote work self-efficacy (Aldabbas et al., 2023). Conversely, when perceived organizational support is low, remote workers feel that the organization does not appreciate their contributions (Jamsen et al., 2022). The organization’s neglect weakens employees’ recognition of their capabilities and reduces their remote work self-efficacy (Li et al., 2018). Chatterjee et al. (2022) indicated that employees who perceive a high level of organizational support increase their focus on organizational goals and tend to meet organizational needs through positive work performance. Therefore, we argue for the following hypothesis:

Hypothesis 4a: Perceived organizational support moderates the relationship between self-control and remote work self-efficacy such that the relationship is stronger when perceived organizational support is higher.

The quality of interactions with supervisors affects employees’ perceptions of self-worth and work significance (Barsness et al., 2005; Kim and Leach, 2021). Especially when employees are under heavy pressure in remote work, interaction with supervisors can serve as an important social support to provide material and spiritual support (Campbell et al., 2013). Evidence shows that interaction with supervisors has a positive impact on employees’ perceptions of organizational equity, work engagement, and work performance (Barsness et al., 2005; Kim and Leach, 2021). Additionally, studies have pointed out that high-quality interactions with supervisors help employees identify and recognize their contributions and cultivate their confidence and sense of responsibility at work, which is an important source of self-efficacy (Herrygers and Wieland, 2017).

We consider that interaction with supervisors moderates the relationship between self-control and remote work self-efficacy. Social cognitive theory suggests that an individual’s self-efficacy may derive from their vicarious experiences of observing others (Bandura, 1978; Staples et al., 1999). From the perspective of vicarious experiences, remote workers have more opportunities to discover similarities with one another or develop similarities through mutual influence when interaction with supervisors is high (McAllister, 1995). Remote workers take their supervisors as examples, regard self-control as an opportunity to keep up with their supervisors, and gain confidence in completing remote work tasks as they learn from their supervisors (Herrygers and Wieland, 2017). On the contrary, when interaction with supervisors is low, remote workers may realize that even with good self-control, it is difficult to establish close relationships with supervisors, which is detrimental to their remote work self-efficacy (Herrygers and Wieland, 2017). A recent study showed that when employees face high work demands, the interpersonal relationships that supervisors provide can buffer employees from stress and enhance their security and confidence (Kim and Leach, 2021). Hence, we suggest the following hypothesis:

Hypothesis 4b: Interaction with supervisors moderates the relationship between self-control and remote work self-efficacy such that the relationship is stronger when interaction with supervisors is higher.

By providing emotional and instrumental help for employees, family support plays an important role in their work (Hui and Lent, 2018; Mehreen and Ali, 2022). Existing research has shown that family support can provide employees with psychological capital that promotes their positive emotions and vitality (Zhu et al., 2017; Mehreen and Ali, 2022). Furthermore, family support has been found to reduce work–family conflict and strengthen work–family balance (Zhu et al., 2017). During remote work, family becomes an important source of social support for employees as they spend more time with their families (Shockley et al., 2021). The stable environment that a family provides can alleviate remote workers’ negative emotions and work stress and prevent their burnout (Restubog et al., 2020).

We indicate that family support moderates the relationship between self-control and remote work self-efficacy. Bandura (2001) stated that situational conditions are also an important factor affecting individual self-efficacy. Unfamiliar situations are more difficult to adapt to and control than other situations, which leads to decreased individual self-efficacy (Bandura, 2001). From the perspective of situational conditions, when family support is high, remote workers have more time and energy to deal with work tasks (Zhu et al., 2017). In this situation, remote workers have a higher degree of control over the family environment and can better apply their self-control ability to the work field, increasing their confidence regarding completing remote work tasks (Mehreen and Ali, 2022). In contrast, when family support is low, remote workers who lack understanding and concern from family members must devote more time and energy to fulfill family obligations (Restubog et al., 2020; Becker et al., 2022). Remote workers in this state cannot control their family environment well and have difficulty playing an active role of self-control during remote work, which can reduce their confidence regarding remote work (Hui and Lent, 2018; Mehreen and Ali, 2022). A study during the pandemic showed that family support can provide a stable environment for remote workers that supplements their energy and boosts their self-esteem and vitality (Galanti et al., 2021). Consequently, we propose the following hypothesis:

Hypothesis 4c: Family support moderates the relationship between self-control and remote work self-efficacy such that the relationship is stronger when family support is higher.

## 3. Method

### 3.1. Participants and procedures

The data was collected in March 2020. Participants were employees who have participated in or are participating in remote work from China. We first issued a recruitment notice via WeChat (a widely used social application in China) to ensure the sample’s validity. In the recruitment notice, we clearly stated the research purpose and indicated that only employees who have participated in or are participating in remote work are eligible to participate in our research. In the questionnaire, we ensured the confidentiality of voluntary participation and response and indicated that participants would receive 20 yuan (about 2.86 dollars) in return for completing the questionnaire.

**Figure 2:**
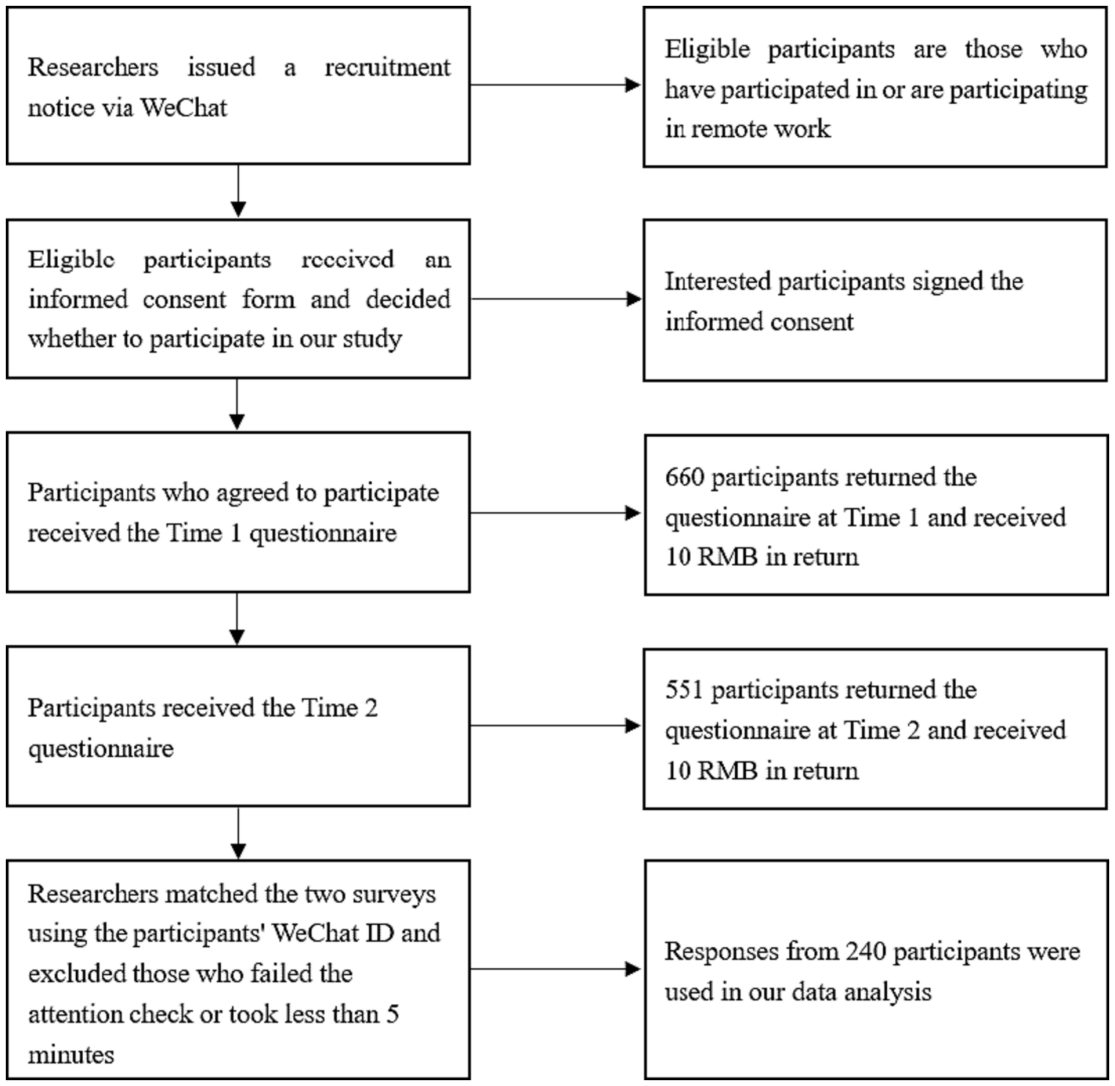
Critical steps in data collection.

The survey was conducted twice, with a one-week interval. We distributed the questionnaires to eligible study participants via WeChat, and participants could reply directly using their smartphones. At Time 1, we collected 660 questionnaires. Participants provided demographic information and rated self-control, perceived organizational support, interaction with supervisors, and family support. At Time 2 (1 week after the Time 1 survey), we collected 551 questionnaires from the same participants at Time 1. Participants rated remote work self-efficacy and remote work effectiveness. We used participants’ unique WeChat IDs to match the two surveys. We also used an attention check to detect and exclude inattentive respondents. We obtained 339 questionnaires (an effective rate of 61.4%) after matching both questionnaires. Excluding those who failed the attention test or took less than 5 min, we finally obtained 240 valid matching questionnaires (the final effective matching rate was 43.5%). Figure **2** summarizes the key steps involved in this process.

The 240 participants comprised 121 males (50.4%) and 119 females (49.5%); the average age was 33.1 years (SD = 6.1); the average level of education was high (84.6% of them had a bachelor’s degree or above); and the average job tenure was 6.8 year (SD = 5.4).

### 3.2. Measures

To ensure the reliability and validity of the measures, we adopted the scales widely used in published authoritative journals. Moreover, to ensure the accuracy of the measures, we created Chinese versions for all measures following the commonly used translation–back translation procedure (Brislin, 1980). All questionnaires were evaluated by the Likert 5-point scoring method (1 = strongly disagree to 5 = strongly agree).

#### 3.2.1. Self-control (T1)

Self-control was measured at Time 1 using the scale from Tangney et al. (2004), which was adapted by Tan and Guo (2008) with a total of 19 items. Sample items included, “I can resist temptation well.” The Cronbach’s alpha was 0.896.

#### 3.2.2. Perceived organizational support (T1)

Perceived organizational support was measured at Time 1 using an eight-item scale developed by Shen and Benson (2016). Sample items included, “My organization cares about my opinions.” The Cronbach’s alpha was 0.952.

#### 3.2.3. Interaction with supervisors (T1)

Interaction with supervisors was measured at Time 1 using the four-item scale from McAllister (1995). Sample items included, “My leaders often initiate work-related interactions with me.” The Cronbach’s alpha was 0.902.

#### 3.2.4. Family support (T1)

Family support was measured at Time 1 using a two-item scale developed by Shen and Benson (2016). Sample items included, “My family is very supportive of my remote work.” The Cronbach’s alpha was 0.833.

#### 3.2.5. Remote work self-efficacy (T2)

Remote work self-efficacy was measured at Time 2 using Staples et al.’s (1999) 16-item scale. Sample items included, “With a written operating manual, I was able to learn (by myself) how to use a computer.” The Cronbach’s alpha was 0.870.

#### 3.2.6. Remote work effectiveness (T2)

Remote work effectiveness was measured at Time 2 using Staples et al.’s (1999) four-item scale. Sample items included “Working remotely is not a productive way to work.” The Cronbach’s alpha was 0.905.

#### 3.2.7. Control variables

We controlled for gender, age, and education in the analyses because these variables have been found to correlate with remote work self-efficacy and remote work effectiveness (Raghuram et al., 2003).

## 4. Results

### 4.1. Common method bias test

This study controls for common method bias by ensuring the clarity of the questionnaire items, enabling participants to fill out the questionnaire anonymously, and collecting data at multiple time points. However, because all items were filled out by employees, we adopted Harman’s single factor test to test the collected data for common method bias. The result indicates that 11 factors with eigen values greater than 1.0, and the first factor extracts contribute to 25.280% (less than 40%). Therefore, common method bias is not identified in this study.

### 4.2. Confirmatory factor analysis

The confirmatory factor analysis (CFA) is conducted to confirm the validity of measures (see Table 1). Results show that a six-factor (i.e., self-control, remote work self-efficacy, remote work effectiveness, perceived organizational support, interaction with supervisors, and family support) measurement model fits the data well: χ^2^ (df = 120) =193.721, *p* < 0.001, CFI = 0.976, RMSEA = 0.051, SRMR = 0.044. We compared the six-factor model with other alternative models, and the results showed that the six-factor model fits the data significantly better than any other alternative model (Bentler and Bonett, 1980). These results indicate that our data support the six-factor measurement model.

**Table 1:** Confirmatory factor analysis results.

Model		χ2	*Df*	RMSEA	CFI	TLI	SRMR
Six-factor model	SC, POS, IWS, FS, RWSE, RWE	193.721	120	0.051	0.976	0.970	0.044
Five-factor model-1	SC, POS + IWS, FS, RWSE, RWE	514.784	125	0.114	0.874	0.845	0.053
Five-factor model-2	SC, POS + FS, IWS, RWSE, RWE	354.511	125	0.087	0.926	0.909	0.072
Five-factor model-3	SC, IWS + FS, POS, RWSE, RWE	333.455	125	0.083	0.932	0.917	0.065
Four-factor model-1	SC + POS + IWS, FS, RWSE, RWE	1038.589	129	0.171	0.705	0.651	0.125
Four-factor model-2	SC + IWS + FS, POS, RWSE, RWE	863.985	129	0.154	0.762	0.718	0.131
Single-factor model	All factors combined	1844.797	135	0.230	0.446	0.372	0.160

*N* = 240; SC, self-control; POS, perceived organizational support; IWS, interaction with supervisors; FS, family support; RWSE, remote work self-efficacy; RWE, remote work effectiveness; TLI, the Tucker–Lewis index; CFI, the comparative fit index; RMSEA, the root-mean-square error of approximation; and SRMR, standardized root mean square residual.

### 4.3. Descriptive statistics

Table 2 shows the descriptive statistics. Results indicate that self-control is positively related to remote work self-efficacy (*r* = 0.249**, *p* < 0.01) and remote work self-efficacy is positively related to remote work effectiveness (*r* = 0.424**, *p* < 0.01). This shows that Hypothesis 1 and Hypothesis 2 have been preliminarily supported.

**Table 2:** Descriptive statistics, correlations, and Cronbach’s alphas.

	*M*	*SD*	1	2	3	4	5	6	7	8	9
1. Gender	0.496	0.501	1								
2. Age	33.125	6.136	−0.037	1							
3. Education	3.092	0.737	−0.090	−0.131*	1						
4. Self-control	3.598	0.674	0.048	0.130*	−0.128*	(0.896)					
5. Remote work self-efficacy	3.888	0.495	0.226**	0.013	−0.055	0.249**	(0.870)				
6. Remote work effectiveness	3.629	0.931	0.104	0.015	−0.087	0.312**	0.424**	(0.905)			
7. Perceived organizational support	3.716	0.949	0.085	0.183**	−0.211**	0.289**	0.360**	0.288**	(0.952)		
8. Interaction with supervisors	3.643	0.986	0.007	0.051	−0.021	0.234**	0.332**	0.296**	0.693**	(0.902)	
9. Family support	4.167	0.947	0.068	−0.010	0.005	0.230**	0.297**	0.308**	0.427**	0.456**	(0.833)

*N* = 240, *p < 0.05; **p < 0.01. Cronbach’s α appears along the diagonal in parentheses.

### 4.4. Hypothesis testing

**Figure 3:**
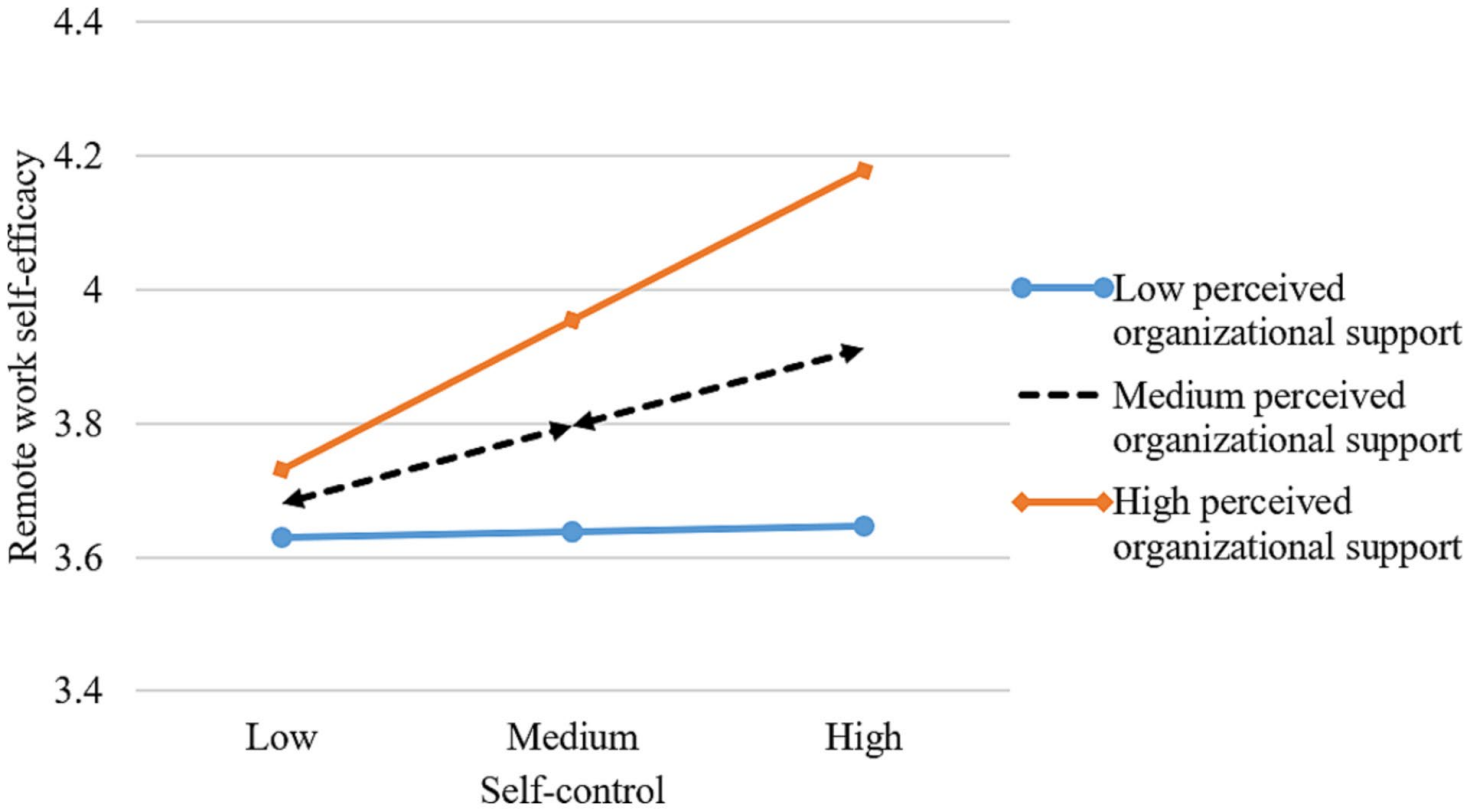
Interaction of self-control and perceived organizational support.

**Figure 4:**
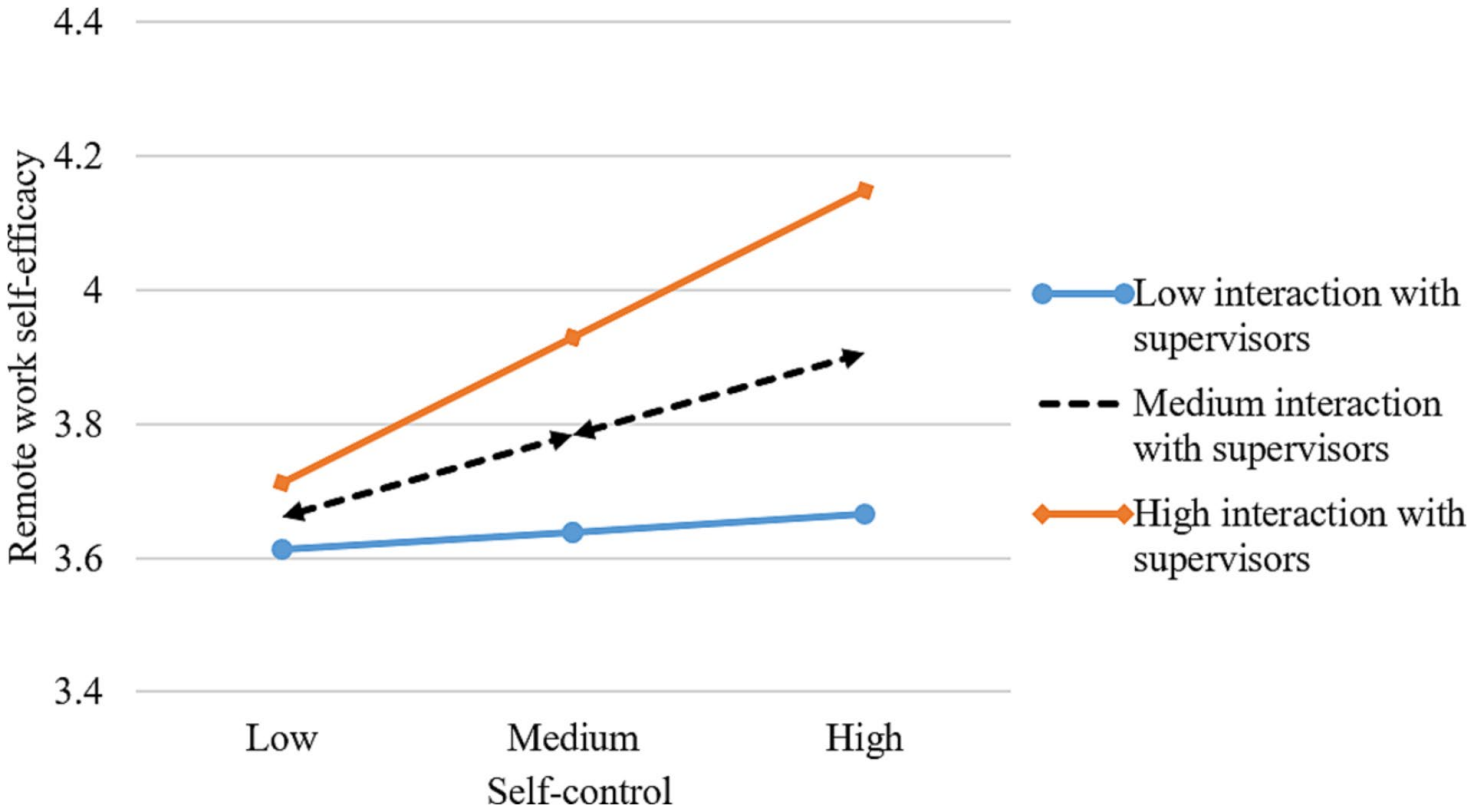
Interaction of self-control and interaction with supervisors.

**Figure 5:**
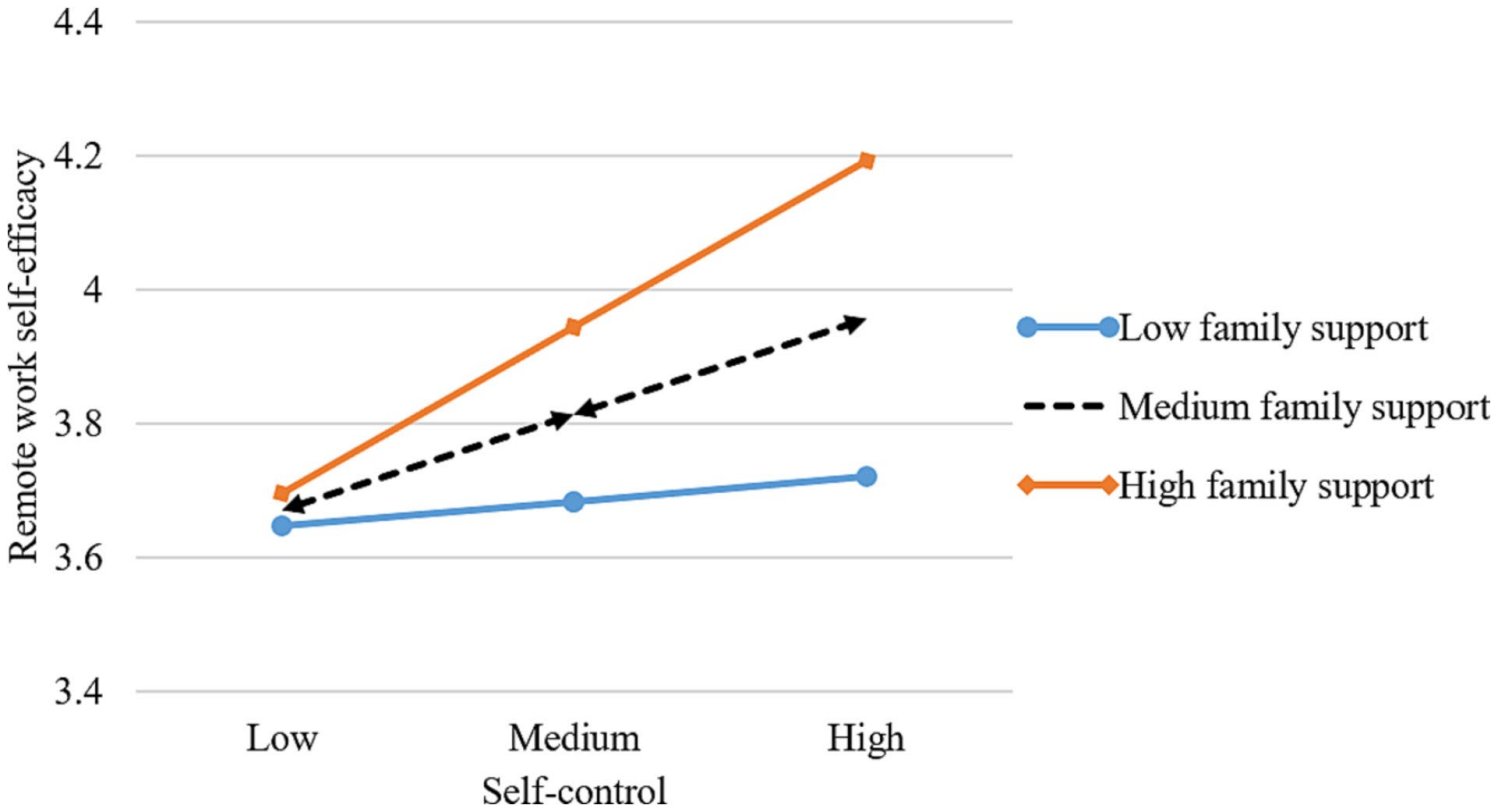
Interaction of self-control and family support.

Table 3 shows the results of the paths analysis. After controlling for the relevant demographic variables (gender, age, education), self-control positively predicts remote work self-efficacy (β = 0.176, p < 0.001), supporting Hypothesis 1. Remote work self-efficacy is positively related to remote work effectiveness (β = 0.787, p < 0.001), supporting Hypothesis 2. We use the bootstrapping method(bootstrap = 1,000) to test the mediating role of remote work self-efficacy. The results show that remote work self-efficacy mediates the relationship between self-control and remote work effectiveness (*β* = 0.121, *p* < 0.001, 95%CI = [0.046, 0.208]), which supports Hypothesis 3. To minimize multi-collinearity, all interaction variables are mean-centered (Aiken and West, 1991). Perceived organizational support (*β* = 0.108, *p* < 0.01), interaction with supervisors (*β* = 0.096, *p* < 0.05), and family support (*β* = 0.106, *p* < 0.01) moderate the effects of self-control on remote work self-efficacy. To determine the nature of the moderating effect, we plotted the interaction using Aiken and West (1991) procedure for computing slopes one standard deviation above and below the mean of the moderator. As Figures **3**–**5** depict, the interaction pattern is consistent with our hypothesis; that is, the relationship between self-control and remote work self-efficacy is stronger when (a) perceived organizational support, (b) interaction with supervisors, or (c) family support is high rather than low. Hence, Hypothesis 4a, 4b, and 4c are supported.

**Table 3:** Results of the paths analysis.

Effects	Paths	Estimate	S. E.	95% CI	Hypotheses test
Direct effects	Self-control → remote work self-efficacy	0.176***	0.046	–	Support H1
	Remote work self-efficacy → remote work effectiveness	0.787***	0.114	–	Support H2
Mediating effect	Self-control → remote work self-efficacy → remote work effectiveness	0.121***	0.042	[0.046, 0.208]	Support H3
Moderating effects	Self-control × perceived organizational support → remote work self-efficacy	0.108**	0.041	–	Support H4a
	Self-control × interaction with supervisors → remote work self-efficacy	0.096*	0.040	–	Support H4b
	Self-control × family support → remote work self-efficacy	0.106**	0.040	–	Support H4c

*N* = 240; *** stands for *p* < 0.001, ** stands for *p* < 0.01, * represents *p* < 0.05. The control variables were gender, age, and education.

## 5. Discussion

It is critical to understand the impact of remote workers’ self-control on their psychological and work performance and how to better play the positive role of self-control (Morgan, 2020; Troll et al., 2022). Based on social cognitive theory, we explore how remote workers’ self-control affects their self-efficacy and effectiveness in remote work, and whether external support from work and family can work synergistically with employees’ internal control. We find that employees’ self-control is positively related to their remote work self-efficacy, which in turn improves their remote work effectiveness. Remote workers with high self-control can gain more work experience and form a better physical and mental state to enhance their remote work self-efficacy. At the same time, through the four processes of selection, cognition, motivation, and emotion, employees’ remote work self-efficacy can ensure that they show higher innovation ability, work quality, and productivity in remote work, which are important manifestations of remote work effectiveness. Furthermore, perceived organizational support, interaction with supervisors, and family support contribute to the positive effect of self-control on remote work self-efficacy. Specifically, in terms of verbal persuasion, perceived organizational support can make employees aware of their value in remote work and enhance their remote work self-efficacy obtained through good self-control. In terms of vicarious experiences, interaction with supervisors can provide employees with the opportunity to learn from their supervisors, make them realize the important role of self-control in remote work, and enhance their belief in completing remote work during in-depth communication with their supervisors. In terms of situational conditions, family support can provide remote workers with an easily adaptable environment that allows them to play the positive role of self-control in remote work under stable conditions and improve their remote work self-efficacy. Thus, our study provides important implications for theory and practice.

### 5.1. Theoretical implications

Our study has several theoretical implications. First, we explore the important role of self-control in remote work, stimulating a broader perspective on self-control at work. Although scholars have thoroughly discussed remote work and self-control (Fan et al., 2020; Shifrin and Michel, 2022), they have inevitably overlooked the impact of self-control on the psychology and behavior of remote workers. With the current prevalence of remote work, elucidating the positive role of self-control in remote work seems more relevant than ever. We echo the call of Troll et al. (2022) to further explore the effect of self-control on the psychology and performance of remote workers under a long-term blockade. Our study not only fills the gap left by previous research but also provides a new perspective for research on remote work.

Second, our study introduces remote work self-efficacy as a mediating variable, expanding the research on the relationship between self-control and remote work effectiveness. Previous research explored the positive role of self-efficacy in the workplace (Howe and Menges, 2022; Makikangas et al., 2022), whereas few studies examined the impact of employees’ self-efficacy on their work outcomes during remote work (Staples et al., 1999). Based on social cognitive theory, domain-specific efficacy is a better predictor of behavior and achievement in a certain context than general efficacy (Bandura, 2001; Makikangas et al., 2022). We refine the remote management framework developed by Staples et al. (1999) and validate the importance of self-efficacy in remote work. Our study not only expands the application of self-efficacy in remote work but also enriches the study of antecedents and consequences of remote work self-efficacy.

Finally, our study examines the moderating effects of perceived organizational support, interaction with supervisors, and family support, revealing the boundary conditions of self-control. Existing research indicated that individuals have limited energy and can be relieved or supplemented by external support to exert greater self-control (Zhang et al., 2015; Orkibi et al., 2018). However, as one of the most accessible external supports, the supplement of social support to self-control is under-explored (Chen et al., 2021; van Zoonen et al., 2021). We consider support from work and family as comprehensively as possible, exploring how the synergy of internal control and external support affects remote workers’ self-efficacy. Our study highlights the complementary role of social support for self-control in remote work and expands the boundary of the relationship between self-control and remote work self-efficacy.

### 5.2. Practical implications

This study also provides several practical implications for organizations. First, it is critical to focus on remote workers’ self-control ability to improve their remote work effectiveness. From the perspective of managers, to ensure the effectiveness of remote workers, it is necessary to set clear and measurable performance goals for remote workers and set a good example for remote workers to properly guide and manage their behavior. From the perspective of employees, to accomplish work more effectively in remote work, remote workers should make detailed work plans for themselves, set appropriate incentives, stay away from temptations in life (e.g., sleeping, playing games, and gossiping), and communicate with team leaders and colleagues promptly (de Ridder et al., 2020).

Second, it is essential to provide remote workers with adequate work and family support to improve their remote work effectiveness. In the domain of work, organizations can provide resources and emotional support for remote workers by conducting technical training, improving benefits, and establishing communication mechanisms (Olson and Olson, 2000; Karis et al., 2016). Supervisors should fully empower remote workers, provide timely feedback, and increase emotional communication to reduce psychological loneliness caused by physical isolation. In the domain of family, organizations should strengthen support and assistance for employees’ families, create family-friendly working conditions, and help them better balance the relationship between family and work in the context of remote work.

### 5.3. Limitations and future research

This study has some limitations, which may provide directions for future research. First, we adopted a multiple-time data collection approach to reduce the concern of common method bias. However, because all measurements were self-reported, there may be some limitations to the findings. Future studies could use multiple-source data to further validate our findings. Second, regarding the sample source, our sample focuses only on Chinese employees, which may limit the generalizability of our findings. Therefore, we call for future studies to further expand the sample by using samples from different countries and different industries to verify our findings. Third, regarding situational factors, we examine the moderating role of three types of social support between self-control and remote work self-efficacy. However, our discussion of social support is not comprehensive and ignores the role of other sources of social support. Future research could explore the moderating role of other social support, such as support from colleagues, support from customers, and support from friends.

## Data availability statement

The raw data supporting the conclusions of this article will be made available by the authors, without undue reservation.

## Ethics statement

Ethical approval was not required for the studies involving humans. The studies were conducted in accordance with the local legislation and institutional requirements. The participants provided their written informed consent to participate in this study.

## Author contributions

LQ: Methodology, Resources, Writing–review–&–editing. YX: Writing–original–draft. BL: Methodology, Resources, Writing–review–&–editing.
